# The Coronavirus Disease (COVID-19) Challenge in Mexico: A Critical and Forced Reflection as Individuals and Society

**DOI:** 10.3389/fpubh.2020.00337

**Published:** 2020-06-26

**Authors:** Claudia Caldera-Villalobos, Idalia Garza-Veloz, Nadia Martínez-Avila, Iván Delgado-Enciso, Yolanda Ortiz-Castro, Griselda A. Cabral-Pacheco, Margarita L. Martinez-Fierro

**Affiliations:** ^1^Molecular Medicine Laboratory, Academic Unit of Human Medicine and Health Sciences, Universidad Autonoma de Zacatecas, Zacatecas, Mexico; ^2^School of Medicine, University of Colima, Colima, Mexico

**Keywords:** COVID-19, SARS-CoV-2, pandemic, health workers, México, coronavirus

## Introduction

Medically speaking we define a crisis as a sudden intensification of the symptoms of a disease (1). According to the Royal Spanish Academy, a crisis is defined as a profound change with important consequences in a process or situation or in the way in which these are appreciated (2). Historically, crisis are opportunities to question the status quo of entire societies; they are moments for reinventing ourselves and thus evolving toward, for example, a better system, a better organized, more tolerant, inclusive, and caring humanity.

## COVID-19: A Global Emergency

Severe acute respiratory syndrome coronavirus 2 (SARS-CoV-2) was identified as the cause of an outbreak of pneumonia of otherwise unknown cause in Wuhan City, Hubei Province, China, in December 2019 (3). Because of the rapid spread of SARS-CoV-2 globally, the World Health Organization declared infection by SARS-CoV-2 a global health emergency on January 20, 2020 (4). According to Johns Hopkins Center for Systems Science and Engineering data, as of June 20, 2020, COVID-19 cases are present in 188 countries. To date, worldwide 8,753,853 cases have been confirmed, of which 463,281 patients died (5). These pictures change daily. In Mexico on June 20, 2020 (phase 3), the statistics showed 175,202 confirmed cases and 20,781 deaths due to COVID-19 (6).

## Mexican Society in The Waiting Room Of SARS-Cov-2

The coronavirus pandemic has quickly become a public health problem and has revealed the deficiencies of our society, not only in Mexico but worldwide. When SARS-CoV-2 arrived in Mexico, it reached a country undergoing one of its most important political-economic transitions, where the economic and health systems were in the midst of reorganization, where it was not clear how the different government agencies would work.

In a context where uncertainty and fear already reigned, with a population that, due to its socio-political background, is characterized by a distrust of its authorities, and which has abundant access to information that is sometimes unreliable (6–8), everything gives rise to new behaviors that limit the ability of the authorities and health personnel to deal with the pandemic (9).

## Health Personnel in Mexico Facing COVID-19

One of the first obstacles faced by health personnel and authorities was a society that did not trust the people taking care of it. Thousands of skeptical Mexicans even claimed that the pandemic would not reach Mexico, driven perhaps by a false a sense of security provided by the country's geographical remoteness from the epicenters of contagion up to that point: China and Italy (10). The first reported case in Mexico was on February 28, 2020 (11). Since its first reported case, the Mexican government gradually increased measures to avoid the increase in the number of cases. These measures included social distancing, closing schools, and the cancellation of sporting events and other gatherings. Almost in parallel (in early March), the Mexican newscasts were flooded with images of people buying scandalous amounts of toilet paper, antibacterial cleaning products, and gels, with the most sophisticated buying medical equipment (12–14). We had not yet entered the period of social distancing and there was already a shortage of supplies. Mexican health personnel throughout the country, from different institutions and levels of care, had to face the early stages of the pandemic without proper protective equipment. Despite this, health personnel continued to provide care to their patients knowing that they did not have the necessary safety provisions, that they were putting their own lives at risk (15, 16). Although this was common knowledge, citizens continued to buy protective medical supplies, even those citizens that were low-risk for the virus (14, 17).

To a certain extent this situation was to be expected, due to lack of preparation for a pandemic, and lack of personal protective equipment (PPE) occurring early in the pandemic. This could have been avoided if government authorities did not underestimate the risk of COVID-19 across Mexico, and instituted adequate response plans early in the pandemic, or even before the virus arrived in Mexico. It is painful but necessary to say that the response and planning by all of the authorities has not been the best, because it seems that more than one governor or municipal president underestimated the situation. Even as healthcare workers protested the lack of PPE and medical equipment, there was no immediate response and healthcare workers continued to work under poor conditions to help those who had contracted COVID-19 (18–20).

The health personnel who have worked overtime since the beginning, who have had to leave their families and friends are now falling ill. It is calculated that up to May 11, 2020, there were 8,544 Mexican health workers infected with SARS-CoV-2, and 111 dead, equivalent to 23.51% of total infections in our country. Of these, 37% are doctors, 41% nurses, 19% other health workers, 2% laboratory workers, and 1% dentist. The number of infected health professionals increased 341.77% in a period of 15 days (20). If we take into account that there are 2.4 doctors for every 1,000 inhabitants in Mexico (21), this means there are approximately 1,317,200 people without access to medical care. If we also take into account that, according to the Mexican First Minister of Health Dr. Jorge Alcocer, there is a deficit of 200,000 doctors and 300,000 nurses to face the pandemic, it is evident that having health personnel sick due to lack of PPE is a situation that Mexico should not have allowed to occur.

In several states of the Mexican Republic, the proportion of confirmed cases that occurred among health personnel was high. Indeed, outbreaks occurring in health institutions even caused their closure (22–24). Numerous doctors and nurses worked and still work in poor conditions, unable to see family and friends, because they are exposed daily to a high risk of suffering contagion, having to work even if they are sick, and in addition, having to bear the risk of bullying/harassment from the general public (9). It is important to consider how log shifts can impact the quality of care, as such multiple studies show extended-duration work shifts significantly increase fatigue and impair performance safety (25–27). This could have as a side effect the inappropriate use of PPE, increasing the risk of contagion.

Another very important aspect was the use of poor quality PPE, which has been a constant throughout the evolution of the pandemic and which has reached such magnitudes that health personnel have seen the need to actively stop and mobilize in protests (28–31).

In other countries, the health workers had a shortage of PPE and/or high exposure to SARS-CoV-2 in health care settings (32, 33). In United States 9,282 (19%) COVID-19 cases were identified as healthcare personnel, while in Spain until March 27, at least 9,444 (14.7%) healthcare workers had been infected with SARS-CoV-2 (34), and more than 6,000 (10%) Italian health workers got sick from COVID-19 (35). These numbers, in these countries, demonstrated that each nation has faced a variety of different obstacles but the common consequence has been a high percentage of healthcare personnel with COVID-19.

## Are Health Workers Heroes, Villains, or Martyrs?

Modes of behavior that we believed to be well-defined have been erased and a social phenomenon has been born: discrimination against health personnel in a climate where everyone yearns to return to their lives in the way they used to be. There have been physical and verbal attacks both inside and outside hospital facilities (36), as well as while they carry out home visits to evaluate patients who, due to their basic diagnosis, had to stay at home. Have also been documented cases of nurses, resident doctors, and other health personnel who have been assaulted on their way home (9, 37), to the point where legal intervention by the authorities was necessary (38). María del Carmen Montenegro, professor at the Faculty of Psychology at the Autonomous University of Mexico, explains: “They (the medical staff) symbolically represent the disease itself and cure it, so the terror that this evil and stigma implies is uncontrollable, which generates the most interesting beliefs as a social process” (39).

## Who is Guilty?

Nobody was prepared for this critical moment and we all could have done more as a population to protect our healthcare workers. We could have respected social distancing adequately, avoided crowded places at the first report of COVID-19, suspended non-essential activities, and complied with the instructions of the health authorities. Questions for the Mexican Government are: Could our authorities have acted more quickly? How effective were the measures and planning that began in January 2020? Should they have done more SARS-CoV-2 diagnostic tests? The answers to these questions may never be known, but we can do better than we are now to improve the working conditions for health personnel and thus help them to preserve the health of us all. We must face this challenge and overcome it in an organized manner with the understanding that our lives are at serious risk.

## Concluding Remarks

We intend for this document to be the beginning point for discussion and for action to improve health institutions in both Mexico and the world and the conditions under which healthcare workers currently work. Above this is an invitation to a change of consciousness and to establish new priorities on the part of our governments. One of the questions on the table must be what will we do to give our health workers an adequate workplace protection? We must think of that we can do from this moment on to create an environment of respect, understanding, and solidarity for those who watch over our health. We must find ways to stop being a society that cares more about improbable wars than ensuring the dignity of life. We must become a society in which, in even in prosperous times, peace and that health are not only the focus of attention when people are dying. We must now ask ourselves what this pandemic means for humanity and how we should live once it ends. That SARS-CoV-2 should not be just another chapter in history, just one more pandemic that plagued humanity—it should become the reconversion point, where the health and lives of all becomes the priority of all nations.

## Author Contributions

CC-V, IG-V, NM-A, ID-E, YO-C, GC-P, and MM-F: made significantly contributions to the conception of the work, to literature search, to writing the manuscript, revised it critically for important intellectual content, approved its final version, and agreed to its submission. All authors contributed to the article and approved the submitted version.

## Conflict of Interest

The authors declare that the research was conducted in the absence of any commercial or financial relationships that could be construed as a potential conflict of interest. The reviewer VCK declared a shared affiliation with one of the authors, ID-E, to the handling editor at time of review.
